# Herbarium‐based measurements are reliable predictors of fresh plant traits in Neotropical Myrtaceae

**DOI:** 10.1002/aps3.70082

**Published:** 2026-09-15

**Authors:** Yacov Kilsztajn, Lázaro Henrique Soares Moraes de Conceição, Thais Vasconcelos, Carolyn Elinore Barnes Proença, Vanessa Graziele Staggemeier

**Affiliations:** ^1^ Programa de Pós‐Graduação em Ecologia Universidade Federal do Rio Grande do Norte Natal 59078−900 Rio Grande do Norte Brazil; ^2^ Departamento de Botânica Universidade de Brasília, Campus Darcy Ribeiro Brasília 70910−900 DF Brazil; ^3^ Department of Ecology and Evolutionary Biology University of Michigan Ann Arbor 48109 Michigan USA; ^4^ University of Michigan Herbarium, University of Michigan Ann Arbor 48108 Michigan USA; ^5^ LEVES ‐ Laboratório de Ecologia Vegetal, Evolução e Síntese, Departamento de Ecologia Universidade Federal do Rio Grande do Norte Natal 59078−900 Rio Grande do Norte Brazil

**Keywords:** flower, fruit, functional traits, leaf, seed, shrinkage effect, water content

## Abstract

**Premise:**

Herbarium specimens are increasingly used to extract morphological traits for ecological and evolutionary studies, yet the effects of tissue desiccation on trait measurements remain poorly understood. Here, we tested two hypotheses: (H1) whether higher tissue water content leads to greater measurement changes after herborization and (H2) whether fresh trait values can be reliably predicted from measurements of herborized specimens.

**Methods:**

We evaluated the reliability of herbarium‐based measurements by comparing fresh and dried traits of leaves, flowers, fleshy fruits, and seeds across 262 individuals representing 133 Neotropical Myrtaceae species. Phylogenetic least squares models and machine learning regressions were used to test H1 and H2.

**Results:**

Leaves and flowers generally shrank after herborization, fruits size metrics tended to increase, and seeds were largely unaffected. Water content was significantly associated with the magnitude of herborization effects in flowers and some leaf and seed traits. Fresh trait values were accurately predicted from measurements of herborized specimens. Prediction errors were lowest for leaf traits, followed by fruits, flowers, and seeds.

**Discussion:**

These results partially support H1 and support H2, indicating that herborized specimens can be reliably used for trait analyses when organ‐specific responses are considered, providing a practical framework to account for potential desiccation bias in functional trait research.

Herbarium specimens have long served as an important resource in plant taxonomy, as botanists rely on dried plant specimens to describe new species and establish taxonomic classifications (Morton, 1981). These preserved materials also are an invaluable historical record of plant diversity and geographical distribution (Mandrioli, 2023; Marín‐Rodulfo et al., 2024). In recent decades, the utility of herbarium data has expanded well beyond taxonomy. Morphological measurements from preserved specimens have increasingly been used to address ecological and evolutionary questions (e.g., Vasconcelos et al., 2019; Jaroszynska et al., 2023; Velásquez‐Puentes et al., 2023; Wilde et al., 2023). This broader application has coincided with the digitization of herbarium collections and the growing accessibility of specimen metadata and high‐resolution images (Soltis, 2017).

Consequently, the combination of herbarium specimens with modern analytical tools is not just improving our understanding of plant biology, but also creating a new paradigm for biodiversity research, creating a wealth of accessible historical data. For instance, recent advances in artificial intelligence and computer vision have enabled the automated extraction of morphological traits from digitized herbarium specimens (e.g., Weaver et al., 2020; Hussein et al., 2021; Guo et al., 2024; Vasconcelos et al., 2026). These innovations have fueled large‐scale studies that integrate thousands of specimens across wide spatial and temporal scales, offering unprecedented opportunities to explore functional trait variation in an ecological and evolutionary context.

However, the use of herbarium specimens in such analyses introduces new challenges related to data accuracy and potential measurement biases. One such bias arises from the desiccation that occurs during specimen preparation. As plant tissues dry they usually shrink, potentially altering the size and shape of morphological traits. Although this phenomenon has been acknowledged, few studies have systematically assessed its impact on trait measurements in herbarium specimens, with most of them focusing primarily on vegetative characters, such as leaf traits (e.g., Blonder et al., 2012; Queenborough and Porras, 2014; Perez et al., 2020). The extent of shrinkage and deformation is likely to vary among plant organs and may depend on their initial water content (Tomaszewski and Górzkowska, 2016). Structures with higher water content, such as flowers and fleshy fruits (Roddy et al., 2023), are expected to shrink more during drying than denser organs, like leaves and seeds.

No study to date has explicitly tested how pressing and drying specimens (hereafter referred to as herborization) affect the accuracy of trait measurements across a range of plant structures. We explore this topic by using the Neotropical clade (sensu Vasconcelos et al., 2017) of the tribe Myrteae (Myrtaceae) as a model group (hereafter Neotropical Myrtaceae), which includes around 50 genera and 2700 species (NMWG et al., 2024). This group is highly variable in terms of vegetative and reproductive traits, including membranaceous to leathery leaves of different shapes and sizes; very small to medium‐sized flowers that are either solitary or arranged in complex inflorescences bearing a few to hundreds of flowers; and a wide variety of fleshy fruits of different sizes, shapes, and textures, as well as different seed morphologies (Valdemarin, 2023; POWO, 2024). Such morphological diversity makes Neotropical Myrtaceae an ideal system to investigate the effects of herborization on trait measurements, while also enabling broader inferences applicable to other plant clades, particularly fleshy‐fruited woody taxa.

We quantify the divergence between trait measurements obtained from herbarium specimens and those measured in fresh material for Neotropical Myrtaceae species. We explicitly test two hypotheses: (H1) the magnitude of size change caused by herborization increases with the water content of the plant organ, and (H2) fresh trait values can be reliably predicted from measurements of herborized specimens.

## METHODS

### Field and herbarium sampling

Field sampling was conducted between November 2024 and November 2025 in 26 localities across Brazil, including areas belonging to three different biomes (Olson et al., 2001): tropical and subtropical grasslands, savannas, and shrublands (*n* = 6); tropical and subtropical moist broadleaf forests (*n* = 14); and desert and xeric shrublands (*n* = 6) (Appendix S1; see Supporting Information with this article). We targeted reproductive individuals of Neotropical Myrtaceae species bearing either flowers and/or mature fruits. Whenever possible, three to five individuals per species were sampled, and two structures per organ were measured for each specimen. In total, we sampled 262 individuals, from 133 species across 14 genera and seven subtribes (Appendix S2).

We quantified a set of morphological traits, including leaf area, leaf length/width ratio, leaf circularity index (calculated as 4 × π × area ÷ perimeter^2^, where values approach 1 for perfectly circular shapes and decrease as shapes become more elongated or irregular), leaf mass per area, petal length, flower length and width (petal‐to‐petal and stamen‐to‐stamen), hypanthium length and width, and fruit and seed length and width (measurements followed Pérez‐Harguindeguy et al., 2016; see Appendix S3 for details on flower measurements). All traits were measured from photographs of fresh structures flattened in an acrylic press with a 5‐cm scale positioned alongside the specimens, using ImageJ (Schneider et al., 2012). Fruit length and width were also measured prior to pressing (except for seven specimens for logistical reasons), as flattening alters the dimensions of mature fleshy fruits. Measurements from pressed fruits were therefore used only to disentangle the effects of pressing and drying on fruit morphology.

After fresh measurements were taken, all samples were subjected to standard herborization procedures (Smith, 1971; Bridson and Forman, 1998). Plant material was carefully arranged between sheets of newspaper and cardboard to ensure uniform flattening and moisture absorption. The layered material was placed in a plant press and evenly compressed using adjustable ropes, with pressure manually adjusted according to organ thickness and tissue consistency to minimize deformation. Pressed specimens were dried in a closed drying chamber at 35–50°C for four to seven days using light bulbs as a heat source. After drying, all morphological measurements were repeated using the same photographic and analytical protocols applied to fresh material. Voucher specimens were prepared in parallel and deposited in the following herbaria: ALCB, CEPEC, IAN, MBM, MICH, MG, PMSP, RB, SPF, UB, and UFRN (acronyms according to Index Herbariorum, 2026). Fresh and dry mass were recorded for each plant structure (leaves, flowers, fruits, and seeds; petioles and peduncles were retained for mass measurements) using a portable balance (Brifit Mini Scale, 0.01 g precision), allowing estimation of water content. Leaf mass per area was calculated using a constant dry mass value for both fresh and dry leaves, divided by leaf area measured in the fresh and dry states, respectively, following Pérez‐Harguindeguy et al. (2016).

### Herborization effect and water content

To quantify the morphological shifts induced by drying, we calculated indices based on paired measurements of fresh and dried materials. First, we estimated the water content (WC) of each plant structure (leaves, flowers, fruits, and seeds) for every sampled specimen as the proportional difference between fresh ($M_{\mathrm{fresh}}$) and dried mass ($M_{\mathrm{dried}}$).

$$\mathrm{WC}=\left(\frac{M_{\mathrm{fresh}}-\;M_{\mathrm{dried}}}{M_{\mathrm{fresh}}}\right)\times 100$$

Second, we quantified the herborization effect ($I_{h}$) for each trait as the ratio between the value measured in dried material ($T_{\mathrm{dried}}$) and its fresh counterpart ($T_{\mathrm{fresh}}$), minus one.

$$I_{h}=\left(\frac{T_{dried}}{T_{fresh}}\right)-1$$

This index represents the proportional change in trait dimensions after herborization, where positive values indicate expansion and negative values indicate shrinkage.

Because fruits are three‐dimensional organs, we applied a two‐step process in order to distinguish between the effect of pressing (mechanical compression) and drying (desiccation). Specifically, the pressing effect was calculated as the ratio between the measurements obtained from newly collected, fresh fruits and the measurements of these fruits pressed (prior to drying), minus one. The drying effect, in turn, was calculated as the ratio between the measurements obtained from the fresh pressed fruits and the desiccated pressed fruits, minus one. By comparing these indices, we were able to partition the total morphological distortion into its mechanical (pressing) and desiccation components. In both cases, positive values indicate expansion, whereas negative values indicate shrinkage.

We tested whether tissue water content explains variation in herborization effect using phylogenetic generalized least squares (PGLS) models fitted to species mean trait values. Analyses were based on the most comprehensive dated phylogeny currently available for Neotropical Myrtaceae (NMWG et al., 2024). Models were fitted separately for leaves, flowers, fruits, and seeds using a maximum likelihood PGLS framework with Pagel's λ estimated from the data, implemented in the R package phylolm (Ho et al., 2016).

To incorporate species lacking phylogenetic placement in the backbone phylogeny (41 species out of 133; Appendix S2), we generated a set of alternative trees by randomly inserting missing taxa within their most likely clades using the R package ape (Paradis and Schliep, 2019). Insertions followed a hierarchical taxonomic procedure: when information on genus sections was available (Appendix S2), species were randomly inserted along branches within the clade defined by congeners belonging to the same section; otherwise, insertions were performed within the genus. This procedure was repeated to generate 100 alternative phylogenetic trees representing uncertainty in species placement. All phylogenetic comparative analyses were repeated across these trees, and parameter estimates were summarized as the mean and standard deviation across trees.

### Predicting fresh trait values

To evaluate whether fresh‐tissue trait values can be predicted from herbarium‐based measurements, we fitted random forest (RF) and gradient boosting (GB) regression models independently for each trait using species‐level mean data, implemented in the R packages randomForest (Liaw and Wiener, 2002) and gbm (Ridgeway, 2013), respectively. We used these two approaches because they differ in their underlying strategies and strengths: RF relies on bagging and is generally robust to overfitting and noise, whereas GB builds models sequentially to optimize predictive performance and can better capture complex patterns, albeit with greater sensitivity to parameter tuning. For each trait, the dataset was randomly partitioned into training (80%) and testing (20%) sets using a fixed seed to ensure reproducibility. Random forest models were fitted with 1000 trees and the number of variables considered at each split was set to the square root of the total number of predictors. Gradient boosting models were fitted with 3000 trees, a maximum tree depth of 3, a learning rate of 0.01, and a subsampling fraction of 0.7. Predictive performance was evaluated on the test data using root mean squared error (RMSE), normalized RMSE (NRMSE; i.e., RMSE divided by the trait range), and the coefficient of determination (*R* ^2^).

To assess whether additional information improved prediction performance, we incorporated phylogenetic information and climatic variables in increasingly complex model formulations: (i) herbarium trait measurements only, (ii) herbarium traits combined with phylogenetic information, and (iii) herbarium traits combined with both phylogenetic and climatic variables. Phylogenetic information was incorporated using phylogenetic eigenvectors derived from phylogenetic trees through principal coordinate analysis of cophenetic distances on the dated tree, using the R package ape (Paradis and Schliep, 2019). Axes that together explained up to 80% of the variance (limited to 10 axes) were retained. To account for uncertainty associated with random species insertion in the phylogeny, analyses were repeated across all alternative trees, and the mean and standard deviation of model performance metrics were calculated across trees (details of species insertion are provided above in the Methods section). Climatic variables were included as species‐level means of mean annual temperature, temperature seasonality (standard deviation of monthly mean temperatures), annual precipitation, and precipitation seasonality (coefficient of variation of monthly precipitation) at specimen collection sites. Geographic coordinates of each specimen were used to extract daily climatic data from the Brazilian Daily Weather Gridded Data dataset (Xavier et al., 2022), from which average climatic variables for the period 2001 to 2025 were calculated. Climatic data extraction and processing were performed in R using the terra and geodata packages (Hijmans et al., 2024, 2026).

## RESULTS

### Herborization effect and water content

**Figure 1:**
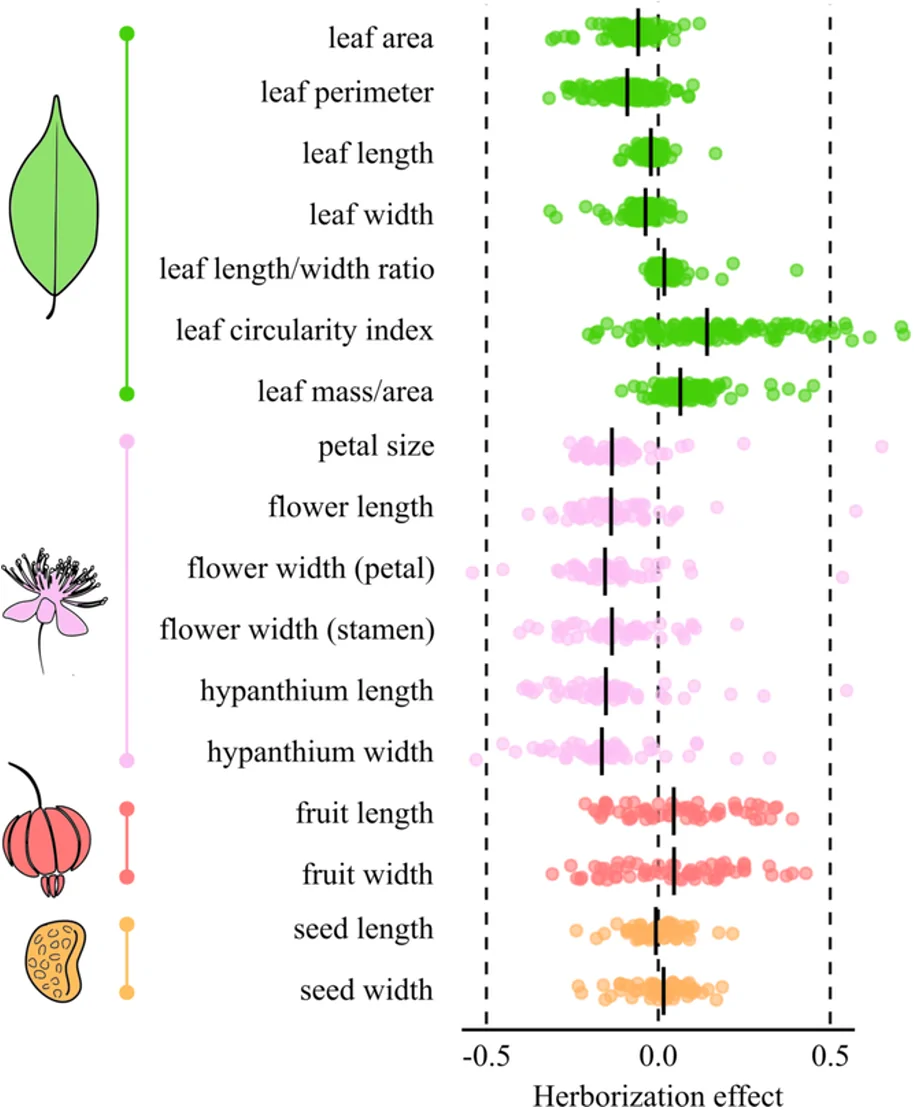
Herborization effect across leaf, flower, fruit, and seed traits. Positive values indicate an increase in trait measurements, whereas negative values indicate a decrease after herborization.

The effects of herborization varied among plant organs (Figure 1). Fruit traits tended to exhibit positive effects, whereas most leaf and floral size traits had negative values. Seed traits had mean effects close to zero. Although reductions were observed in leaf size metrics, shape‐related traits, such as length/width ratio and circularity index, as well as leaf mass per area, generally increased after herborization. Variation in herborization effects among species was comparatively low for leaf traits and more pronounced for fruit traits, with floral and seed traits having intermediate levels of variation. When the herborization effect for fruits was decomposed into pressing and drying components (Appendix S4), fruit length and width increased during pressing but subsequently decreased during drying, resulting in the net positive effect observed.

**Figure 2:**
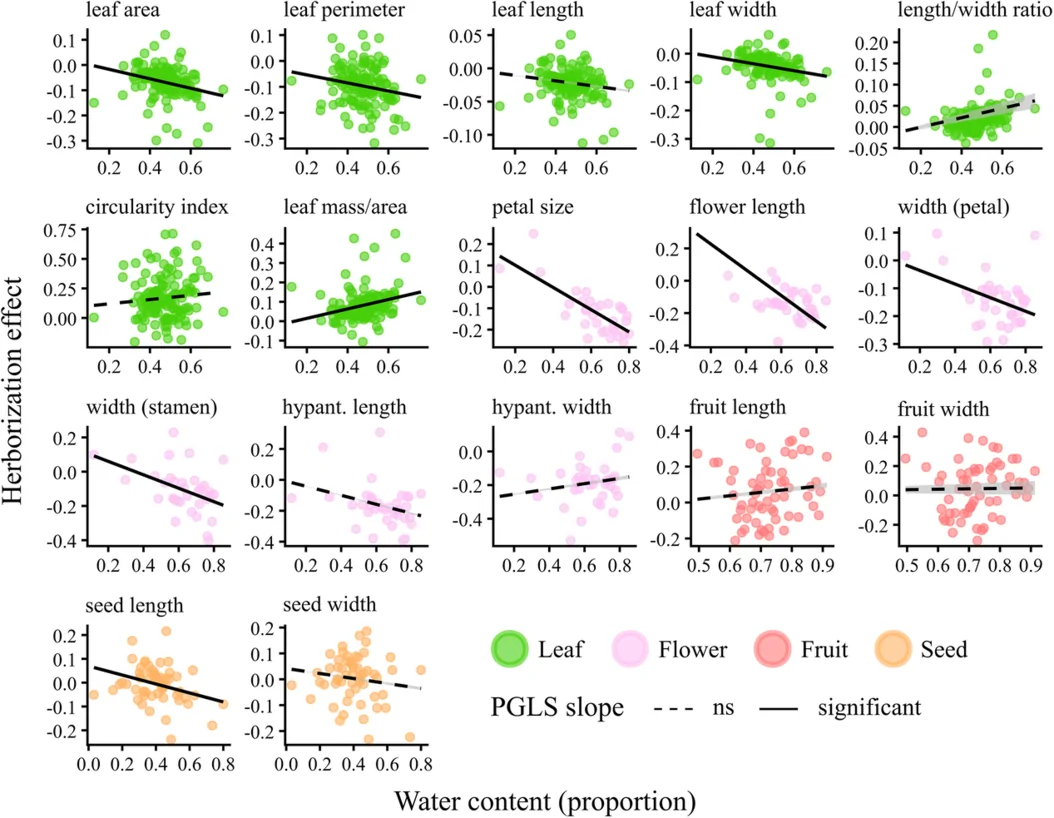
The effect of water content on the herborization effect for leaf, flower, fruit, and seed traits. Each point represents the mean trait value for a species. Lines indicate slopes estimated by phylogenetic generalized least squares (PGLS) models for each trait; dashed lines represent nonsignificant relationships.

Water content explained a variable proportion of the herborization effect across plant organs (Figure 2, Table 1). Significant associations between water content and the herborization effect were generally negative, indicating greater shrinkage in structures with higher water content. The only exception was leaf mass per area, which showed a positive relationship, indicating that leaves with higher water content tended to exhibit greater increases in leaf mass per area after herborization. For fruits, no significant relationships were detected (Figure 2, Table 1); however, trends were positive, suggesting that the herborization effect tends to result in greater expansion in fruits with higher water content. The herborization effect on flower hypanthium dimensions and leaf shape–related traits showed weak or nonsignificant relationships with water content (Figure 2, Table 1).

**Table 1:** Summary statistics for phylogenetic generalized least squares (PGLS) models across the 100 alternative phylogenetic trees (mean and SD) testing the relationship between trait herborization effect and water content for each plant organ.

Trait	Slope (mean)	Slope (SD)	*P*‐value (mean)	*R* ^2^ (mean)	*R* ^2^ (SD)
Flower width (petal)	−0.24384026	0.01449239	0.01207981	0.19691979	0.05530127
Flower width (stamen)	−0.39214905	0.02181113	0.00669024	0.21556878	0.04568122
Hypanthium width	0.15725916	0.01339637	0.26586882	0.05434366	0.04193678
Hypanthium length	−0.29219894	0.00660988	0.05363341	0.13210540	0.04222062
Flower length	−0.79537896	0.00046846	0.00004075	0.40418720	0.00109989
Petal size	−0.52045398	0.00281888	0.00000044	0.56758677	0.00653224
Fruit width	0.02853937	0.05136223	0.85774145	0.32429006	0.02518732
Fruit length	0.18628523	0.01515661	0.41646934	0.17719487	0.01664554
Leaf area	−0.18762177	0.00526459	0.00137893	0.19731026	0.02569370
Leaf circularity index	0.18048391	0.00143589	0.29584891	0.00986783	0.00583313
Leaf length	−0.04137087	0.00195487	0.13170018	0.09599497	0.02422306
Leaf length/width ratio	0.11138431	0.02338754	0.15559992	0.07683805	0.08232525
Leaf mass per area	0.24305401	0.00645281	0.00125285	0.21545671	0.01657239
Leaf perimeter	−0.15578900	0.00420026	0.03004510	0.05195782	0.02342023
Leaf width	−0.12282659	0.00276052	0.00853460	0.05840962	0.01347688
Seed width	−0.09666408	0.00729293	0.26367383	0.19616219	0.02061867
Seed length	−0.18806221	0.00391914	0.01303492	0.21106978	0.01357359

### Predicting fresh trait values

**Figure 3:**
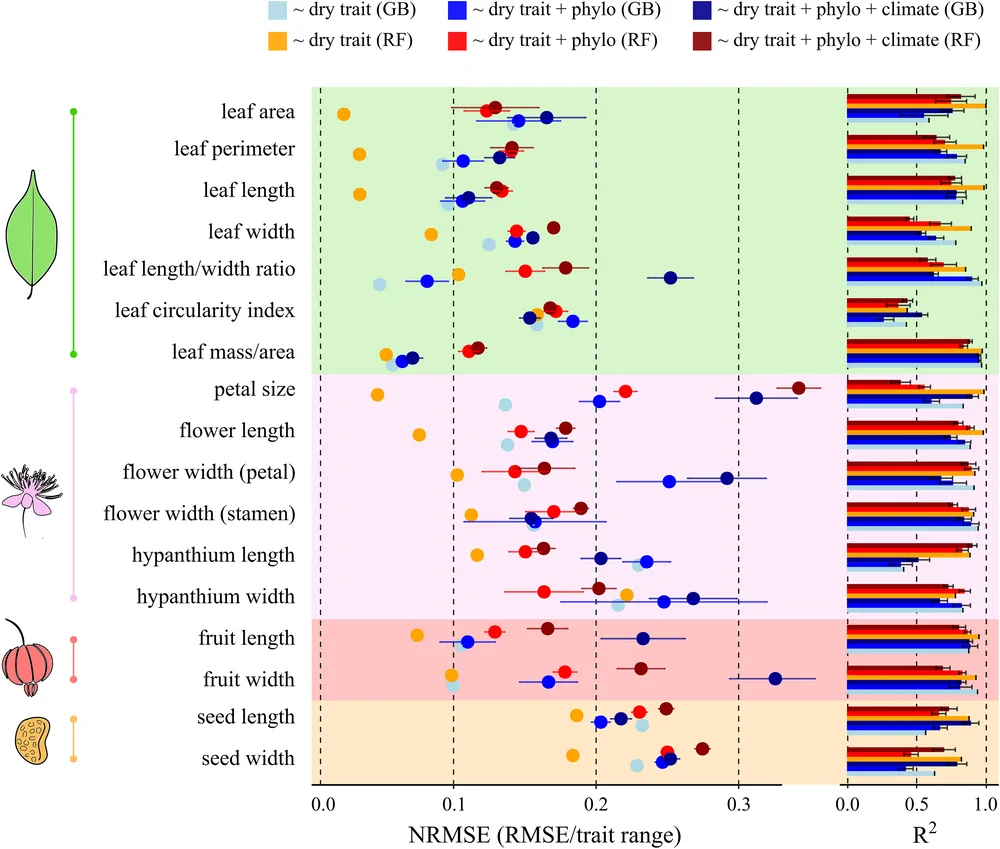
Comparison of model performance for gradient boosting (GB) and random forest (RF) regression models predicting fresh‐tissue trait values from herbarium‐based measurements and additional predictors. Panels show normalized root mean squared error (NRMSE; left) and the coefficient of determination (*R* ^2^; right) for each trait. Confidence intervals represent the standard deviation across analyses using 100 alternative phylogenetic trees. Models differ in predictor sets: herbarium‐based measurements only (~dry trait), herbarium traits with phylogenetic information (~dry trait + phylo), and herbarium traits with both phylogenetic information and climatic variables (~dry trait + phylo + climate). Trait categories (leaf, flower, fruit, and seed) are indicated by icons on the left.

Models based on herbarium measurements successfully predicted fresh trait values across most plant structures (Figure 3, Appendix S5). For most traits, the best‐performing model (lowest NRMSE and higher *R* ^2^) was the one using only dried herbarium measurements. In general, RF performed better, whereas GB had better predictive performance for leaf circularity index, hypanthium width, and seed length and width. In a few cases, models including additional predictors performed best: the phylogeny‐informed model for leaf width and leaf circularity index, and the model including both phylogenetic and climatic predictors for hypanthium and fruit width (Figure 3, Appendix S5).

Leaf traits showed high predictive performance in the selected models (Figure 3, Appendix S5). Leaf size metrics and leaf mass per area were accurately predicted (*R* ^2^ = 0.89–0.99; NRMSE = 0.02–0.08), whereas leaf shape metrics showed lower predictive performance (*R* ^2^ = 0.43–0.87; NRMSE = 0.12–0.16). Floral traits were also accurately predicted (*R* ^2^ = 0.95–0.98; NRMSE = 0.05–0.11), with the exception of hypanthium measurements, which showed comparatively lower predictive performance (*R* ^2^ = 0.85–0.91; NRMSE = 0.12–0.15). Fruit traits were accurately predicted from herbarium measurements (*R* ^2^ = 0.92–0.95; NRMSE = 0.07–0.11). In contrast, seed traits showed lower predictive performance (*R* ^2^ = 0.82–0.88; NRMSE = 0.18–0.19).

## DISCUSSION

This study evaluated how herborization affects morphological trait measurements and whether fresh trait values can be reliably inferred from herbarium specimens across multiple organs in Neotropical Myrtaceae, with potential applicability to other fleshy‐fruited woody taxa. We hypothesized that (H1) morphological change during herborization would increase with organ water content and that (H2) fresh trait values could be accurately predicted from measurements of herborized specimens. Our findings provide partial support for the first hypothesis, as water content was strongly associated with herborization effects primarily in floral traits but showed weaker or nonsignificant relationships in leaves, fruits, and seeds. In contrast, the second hypothesis was strongly supported: measurements obtained from herborized specimens accurately predicted fresh trait values across most traits. Together, these results indicate that although herborization can alter some morphological measurements, particularly in water‐rich tissues, herbarium specimens may retain substantial quantitative information that can be used to estimate fresh trait values.

### Herborization effect and water content

Herborization effects varied among plant structures. The reductions observed in leaf dimensions are consistent with previous studies showing that drying commonly causes leaf shrinkage (Blonder et al., 2012; Tomaszewski and Górzkowska, 2016). The increase in leaf mass per area after drying also aligns with earlier findings that specific leaf area (the inverse of leaf mass per area) decreases following herborization due to reductions in leaf area during desiccation (Queenborough and Porras, 2014; Perez et al., 2020). Because both metrics are calculated using leaf dry mass (Pérez‐Harguindeguy et al., 2016), a decrease in leaf area necessarily leads to higher leaf mass per area and lower specific leaf area. The magnitude of shrinkage observed here is comparable to that reported for woody and evergreen taxa (approx. 10%; Blonder et al., 2012), which is particularly relevant given the predominance of woody evergreen species in Myrtaceae. Water content was generally negatively associated with the magnitude of herborization effects in size‐related leaf traits, but these relationships were weak, suggesting that structural properties such as lignin content (Juneau and Tarasoff, 2012) may play a more important role in determining leaf shrinkage.

Despite the general shrinkage of leaf dimensions, shape‐related traits such as length/width ratio and circularity index increased after herborization. This pattern suggests that desiccation affects leaf axes unevenly, resulting in differential shrinkage rather than uniform contraction. Therefore, although herbarium specimens provide reliable estimates of absolute leaf size, caution is needed when interpreting shape‐based traits that are sensitive to proportional changes. Tomaszewski and Górzkowska (2016) similarly reported that leaf shape is often not fully preserved after drying. In most species they examined, leaves became narrower, consistent with our observed increase in length/width ratio. However, the magnitude of these alterations appeared highly species‐specific, which the authors attributed to anatomical differences, particularly in secondary venation patterns and lignin content. Notably, that study was based on relatively limited taxonomic sampling (22 species), highlighting the need for broader comparative assessments across diverse lineages.

Extending this framework beyond leaves, our study shows that floral traits exhibit similar but often stronger size reductions. Flowers tend to be less lignified and are typically short‐lived organs not adapted for structural persistence, which likely explains their greater sensitivity to desiccation (Ievinsh, 2023; Roddy et al., 2023). Consistent with this, floral traits showed the strongest and most consistent relationships with water content, with species exhibiting higher floral water content experiencing greater shrinkage during drying. This pattern supports the expectation that tissues with a higher proportion of hydrated cells are particularly sensitive to desiccation.

Seed traits, in contrast, exhibited relatively limited dimensional changes. This may reflect the fact that dehydration is an intrinsic component of seed maturation, meaning their tissues are developmentally adapted to water loss (Bewley et al., 2012). Although most Myrtaceae seeds are recalcitrant and retain relatively high water content compared with seeds in many other plant clades (Wyse and Dickie, 2017), the magnitude of shrinkage observed here was small, suggesting that seed measurements from herbarium specimens may still be broadly reliable. Consistent with this interpretation, seed traits showed intermediate relationships with water content, indicating that hydration contributes to variation in shrinkage but is not its primary driver.

Fruit traits showed no clear relationship between water content and herborization effects. The absence of a clear water‐content signal in fruits may reflect the strong mechanical effects associated with specimen preparation. This indicates that dimensional changes in fruits are largely driven by mechanical flattening prior to desiccation rather than by water loss alone. Fruits in the tribe Myrteae are predominantly fleshy berries with high water content, often ranging from 71% to 94% of pulp mass (Galetti et al., 2011). Pressing these three‐dimensional structures during specimen preparation can therefore substantially distort their morphology, particularly in mature fruits, demonstrating that herborization effects may reflect multiple processing steps rather than drying alone.

To our knowledge, this study is the first to explicitly disentangle and quantify the separate contributions of pressing and drying to herborization‐related measurement bias. While this distinction may be less critical for relatively flat structures such as leaves (e.g., Blonder et al., 2012; Juneau and Tarasoff, 2012; Queenborough and Porras, 2014; Tomaszewski and Górzkowska, 2016; Perez et al., 2020), it is particularly important for fleshy and three‐dimensional organs. These results therefore indicate that fruit traits derived from herbarium specimens require particular caution, especially in clades characterized by fleshy fruits with high water content.

### Predicting fresh trait values

Fresh trait values were reliably predicted from measurements of herborized specimens across most plant structures. In most cases, models using only dried trait measurements performed best, indicating that dimensional changes caused by herborization are sufficiently consistent to allow accurate reconstruction of fresh values without additional predictors.

Predictive performance was highest for leaf size traits, reinforcing previous findings that herbarium specimens provide reliable estimates of leaf dimensions (Blonder et al., 2012). In contrast, leaf shape metrics were less accurately predicted, likely reflecting uneven deformation during drying that alters proportional relationships among leaf axes (Tomaszewski and Górzkowska, 2016). Floral traits also showed strong predictive performance overall, although some structures such as the hypanthium were slightly more sensitive to distortion during specimen preparation. Predictions for fruits and seeds were generally robust but somewhat less accurate, probably reflecting the three‐dimensional structure of these organs and the mechanical effects associated with pressing these tissues.

Additional predictors contributed little to model performance. Phylogenetic information improved predictions for only a few traits, and climatic variables rarely reduced prediction error. Together, these results indicate that dried trait measurements alone usually capture most of the information needed to estimate fresh values. This pattern aligns with the conclusions of Blonder et al. (2012), who found that evolutionary history and climate played minor roles when predicting leaf shrinkage.

### Caveats and future research

Our study has some limitations that should be considered when interpreting the results. First, our sampling primarily addresses interspecific variation. We did not explicitly examine intraspecific variation, and future studies should assess whether the patterns identified here hold not only among species but also within species. Second, we focus exclusively on the effects of herborization (i.e., pressing and drying) on trait measurements. Long‐term storage of herbarium specimens may further influence structural dimensions. Although it is reasonable to assume that most differences between fresh and herbarium material arise during herborization rather than storage, prolonged storage could amplify these differences and potentially affect the predictive performance of our models. The effects of long‐term storage have not been explicitly addressed in previous similar studies (e.g., Blonder et al., 2012; Juneau and Tarasoff, 2012; Queenborough and Porras, 2014; Tomaszewski and Górzkowska, 2016; Perez et al., 2020) and therefore remain an open question. The ongoing digitization of herbarium collections offers a promising opportunity to investigate this issue. For example, traits could be measured from both digital images and their corresponding physical specimens, enabling comparisons across different time points and providing a proxy for assessing storage effects on trait measurements.

### Concluding remarks

Our findings support the potential of herbarium collections as large‐scale sources of biological trait data, while highlighting important limitations. The ability to infer fresh morphological traits from preserved specimens suggests that herbarium material can contribute to emerging collectomics approaches (Bucher et al., 2025), which aim to extract and integrate biological information from natural history collections using automated and computational tools. However, these inferences should be made with caution, as our analyses focus on the effects of herborization and do not account for potential changes associated with long‐term storage, which remain poorly understood. Because collecting fresh trait data in the field often requires substantial logistical effort and financial investment, particularly in remote or species‐rich regions, herbarium specimens offer a valuable complementary resource for expanding trait datasets across taxa, space, and time (Davis, 2023). Nevertheless, the applicability of this approach may vary among structures and traits, and further work is needed to assess its robustness across different contexts, including intraspecific variation and storage‐related effects. Overall, our results contribute to the growing role of collectomics in leveraging biological collections for large‐scale ecological and evolutionary research (Sigwart et al., 2025), while emphasizing the need for careful interpretation and further validation.

## AUTHOR CONTRIBUTIONS

Y.K., T.V., and V.G.S. conceived and designed the research. All authors collected the data. Y.K. performed the data analysis and wrote the manuscript. V.G.S. acquired the funding and supervised the study. All authors approved the final version of the manuscript.

## Supporting information


**Appendix S1:** Geographic distribution of sampled specimens across Brazil. Sampling localities within a 0.3° radius were aggregated for visualization purposes.

**Appendix S2:** Sampled specimens with corresponding herbarium vouchers and information on whether each species is included in the most recently published phylogeny for Neotropical Myrtaceae (NMWG et al., 2024).

**Appendix S3:** Details of flower trait measurements: (a) petal size, (b) flower length, (c) flower width (petal‐to‐petal), (d) flower width (stamen‐to‐stamen), (e) hypanthium length, (f) hypanthium width.

**Appendix S4:** Box plot showing the separate effects of fruit pressing and drying on length and width measurements.

**Appendix S5:** Comparison of model performance for gradient boosting (GB) and random forest (RF) regression models predicting fresh‐tissue trait values from herbarium‐based measurements. Summary performance metrics are reported for each trait across all models and across the 100 alternative phylogenetic trees (mean and SD). Models differed in predictor sets: herbarium‐based measurements only (~dry trait), herbarium traits plus phylogenetic information (~dry trait + phylo), and herbarium traits plus both phylogenetic information and climatic variables (~dry trait + phylo + climate).

**Appendix S6:** Species mean data including fresh and dry trait measurements, fresh and dry structure weights, herborization effects, and mean climatic variables.

## Data Availability

The data supporting the findings of this study are available in the Supporting Information of this article (Appendix S6). Additionally, all data and code are openly accessible at https://github.com/ykilsztajn/fresh_dry_myrtaceae.
