# Supplementary material for: Herbarium‐based measurements are reliable predictors of fresh plant traits in Neotropical Myrtaceae
Source: Appl Plant Sci. 2026 Sep 15:e70082. Online ahead of print. doi: 10.1002/aps3.70082 (PMC13576477; doi:10.1002/aps3.70082)

DOI 10.1002/aps3.70082

APPENDIX S3. Details of flower trait measurements: (a) petal size, (b) flower length, (c) flower width (petal-to-petal), (d) flower width (stamen-to-stamen), (e) hypanthium length, (f) hypanthium width.

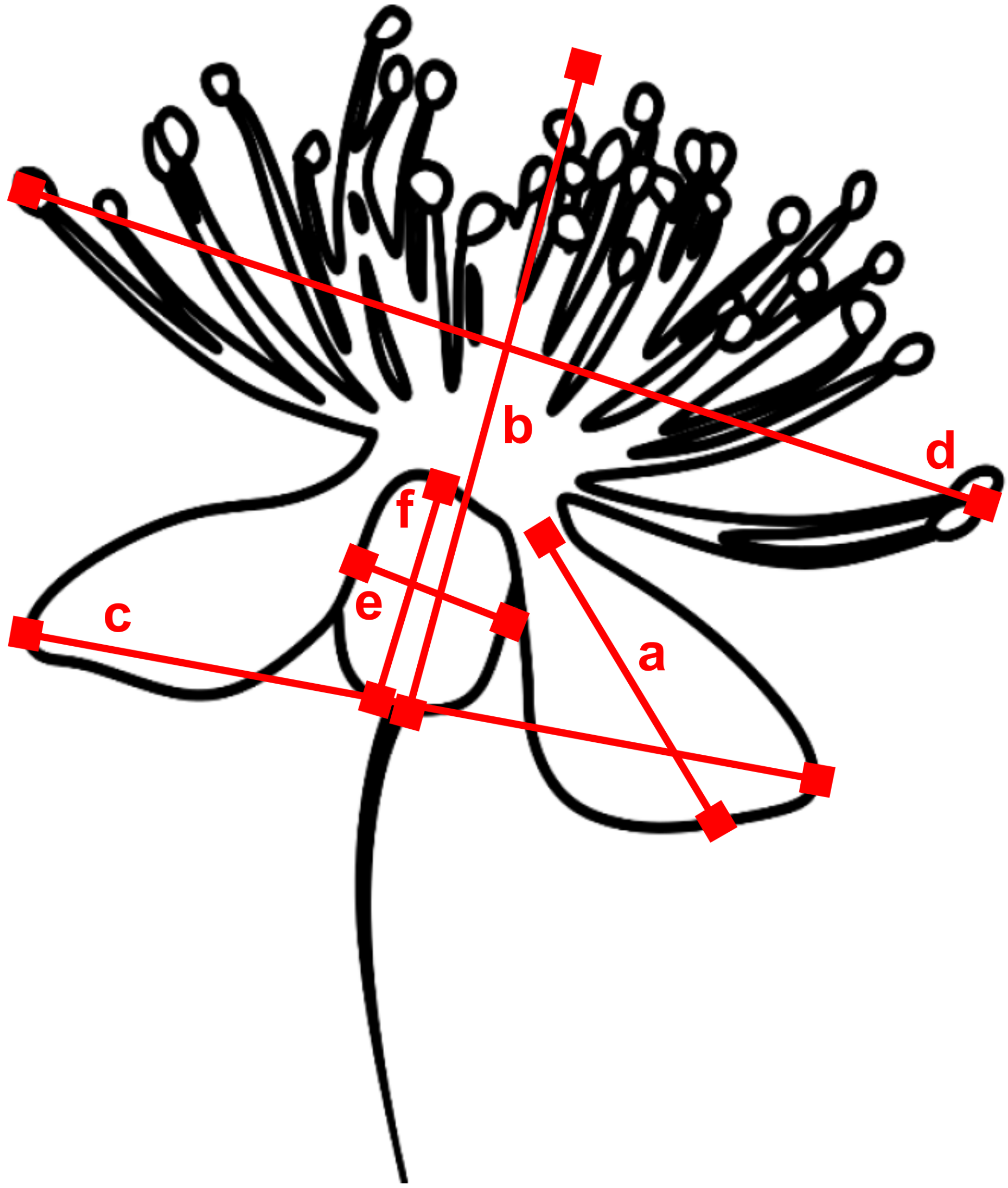

Supplement: Supplementary file 1 [file APS3-9999-e70082-s001.pdf]
