# Supplementary material for: Herbarium‐based measurements are reliable predictors of fresh plant traits in Neotropical Myrtaceae
Source: Appl Plant Sci. 2026 Sep 15:e70082. Online ahead of print. doi: 10.1002/aps3.70082 (PMC13576477; doi:10.1002/aps3.70082)

DOI 10.1002/aps3.70082

APPENDIX S4. Box plot showing the separate effects of fruit pressing and drying on length and width measurements.

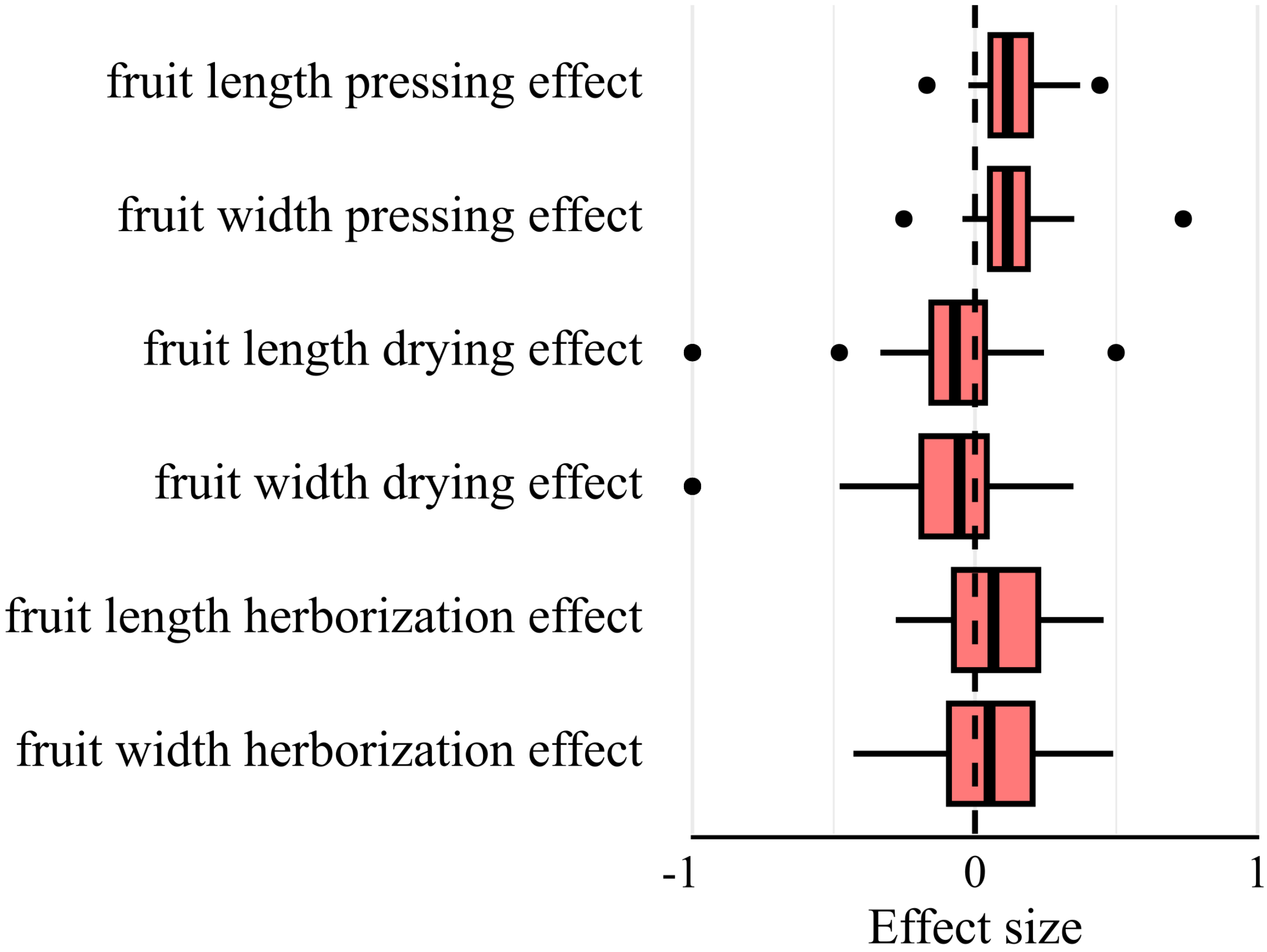

Supplement: Supplementary file 2 [file APS3-9999-e70082-s002.pdf]
