# Supplementary material for: Herbarium‐based measurements are reliable predictors of fresh plant traits in Neotropical Myrtaceae
Source: Appl Plant Sci. 2026 Sep 15:e70082. Online ahead of print. doi: 10.1002/aps3.70082 (PMC13576477; doi:10.1002/aps3.70082)

DOI 10.1002/aps3.70082

APPENDIX S1. Geographic distribution of sampled specimens across Brazil. Sampling localities within a 0.3° radius were aggregated for visualization purposes.

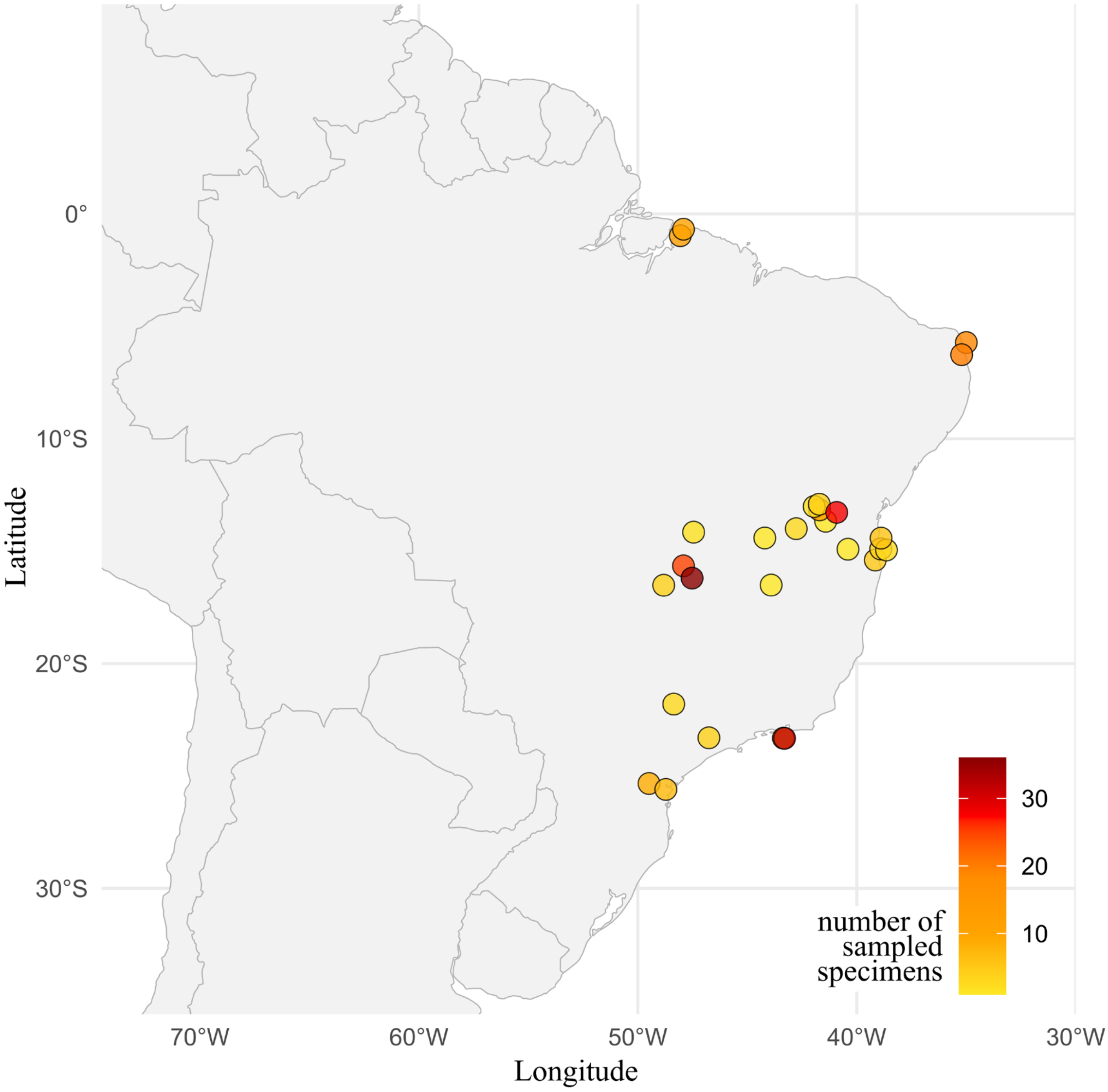

Supplement: Supplementary file 3 [file APS3-9999-e70082-s003.pdf]
